# Regulation of SMC traction forces in human aortic thoracic aneurysms

**DOI:** 10.1007/s10237-020-01412-6

**Published:** 2021-01-15

**Authors:** Claudie Petit, Ali-Akbar Karkhaneh Yousefi, Olfa Ben Moussa, Jean-Baptiste Michel, Alain Guignandon, Stéphane Avril

**Affiliations:** 1 Mines Saint-Etienne, Université de Lyon, INSERM, U 1059 SAINBIOSE, 42023 Saint-Etienne, France; 2 Université Jean Monnet, Université de Lyon, INSERM, U 1059 SAINBIOSE, 42023 Saint-Etienne, France; 3 UMR 1148, Laboratory for Translational Vascular Science, Xavier Bichat Hospital, Inserm and Paris 7- Denis Diderot University, 75018 Paris, France

**Keywords:** Cell biomechanics, Ascending thoracic aortic aneurysm (ataa), Mechanotransduction, Smooth muscle cells (smc), Traction force microscopy (tfm), Single cell

## Abstract

Smooth muscle cells (SMCs) usually express a contractile phenotype in the healthy aorta. However, aortic SMCs have the ability to undergo profound changes in phenotype in response to changes in their extracellular environment, as occurs in ascending thoracic aortic aneurysms (ATAA). Accordingly, there is a pressing need to quantify the mechanobiological effects of these changes at single cell level. To address this need, we applied Traction Force Microscopy (TFM) on 759 cells coming from three primary healthy (AoPrim) human SMC lineages and three primary aneurysmal (AnevPrim) human SMC lineages, from age and gender matched donors. We measured the basal traction forces applied by each of these cells onto compliant hydrogels of different stiffness (4, 8, 12, 25 kPa). Although the range of force generation by SMCs suggested some heterogeneity, we observed that: 1. the traction forces were significantly larger on substrates of larger stiffness; 2. traction forces in AnevPrim were significantly higher than in AoPrim cells. We modelled computationally the dynamic force generation process in SMCs using the motor-clutch model and found that it accounts well for the stiffness-dependent traction forces. The existence of larger traction forces in the AnevPrim SMCs were related to the larger size of cells in these lineages. We conclude that phenotype changes occurring in ATAA, which were previously known to reduce the expression of elongated and contractile SMCs (rendering SMCs less responsive to vasoactive agents), tend also to induce stronger SMCs. Future work aims at understanding the causes of this alteration process in aortic aneurysms.

**Supplementary information:**

The online version of this article (10.1007/s10237-020-01412-6) contains supplementary material, which is available to authorized users.

## Introduction

Ascending Thoracic Aortic Aneurysms (ATAA) remain among the most critical cardiovascular diseases. They constitute significant weakening of the aortic wall and increase the risk of dissection or rupture, which constitute the most fatal complication (Isselbacher 2005; Goldfinger et al. 2014). Currently, the standard criterion for surgical interventions is the aortic diameter, measured by echography or CT-scan (Isselbacher 2005; Goldfinger et al. 2014). Nevertheless, a significant number of dissections or ruptures were reported for small aneurysms (Hagan et al. 2000). Therefore, the diameter appears insufficient to characterize rupture risk in the aortic wall, and several supplemental criteria, accounting for biomechanical properties of the aortic wall, have been proposed recently (Choudhury et al. 2009; Hayashi et al. 2012; Humphrey et al. 2015; Trabelsi et al. 2015; Duprey et al. 2016; Farzaneh et al. 2019). Mechanobiology of ATAA also appears to be determinant. The aorta, like any other biological tissue, is composed of cells embedded in an Extracellular Matrix (ECM). Cells synthesize and remodel this ECM according to stimuli they receive or sense from their environment. Mis-sensing of mechanical stimuli, such as stress, strain or stiffness, was typically shown to play a major role in ATAA (Humphrey et al. 2015; Milewicz et al. 2016).

The aorta is composed of three layers, namely (from internal to external) the intima, the media, and the adventitia. The media typically represents 2/3 of the whole thickness of the wall and contains the Smooth Muscle Cells (SMCs) that have a key role in both the passive and active mechanical response of the aortic tissue (Lacolley et al. 2012; Humphrey et al. 2015; Michel et al. 2018). SMCs are normally highly contractile and may react to both biochemical (Thyberg et al. 1990, 1997; Reusch et al. 1996; Tran et al. 2006; Wang and Lin 2007; Chen et al. 2015) and mechanical stimuli from the surrounding ECM (Owens et al. 1981, 2004; Rubbia and Gabbiani 1989; Humphrey 2002; Arribas et al. 2006; Tsamis et al. 2013; Mao et al. 2015).

Nevertheless, in the ATAA pathology, genetics (Schildmeyer et al. 2000; Chen et al. 2007; Guo et al. 2007; Kuang et al. 2012; Gillis et al. 2013; Regalado et al. 2015; Papke et al. 2015; Milewicz et al. 2016; Brownstein et al. 2017), hemodynamics (Choudhury et al. 2009; Pasta et al. 2013), or biomechanics (Isselbacher 2005; Humphrey et al. 2015; Brownstein et al. 2017) may alter the contractile function of aortic SMCs. This may result in increased apoptosis (Li and Xu 2007; Riches et al. 2013; Mao et al. 2015) and phenotype switching from a mature quiescent and contractile phenotype towards a synthetic one (Thyberg et al. 1990; Mecham and Schwartz 1995; Humphrey 2002; Owens et al. 2004). Synthetic SMCs have the ability to synthesize structural proteins like collagen and glycosaminoglycans (GaGs) and to remodel the ECM (Mecham and Schwartz 1995; Humphrey 2002; Cox and Erler 2011; Lacolley et al. 2012; Hayashi et al. 2012; Hong et al. 2014; Humphrey et al. 2015). They are present normally during early stages of development: they are able to proliferate and migrate in order to build tissue (Thyberg et al. 1990; Humphrey 2002). In adulthood, they remodel the aortic structure under pathological conditions through ECM production and degradation (Rubbia and Gabbiani 1989; Owens et al. 2004; Arribas et al. 2006; Hayashi et al. 2012; Bellini et al. 2014; Papke et al. 2015). Moreover, previous studies reported an alteration of the contractile apparatus, suggesting that an increasing number of synthetic SMCs decreases the responsiveness to vasoactive agents or stimuli (Rubbia and Gabbiani 1989; Schildmeyer et al. 2000; Owens et al. 2004; Chen et al. 2007; Humphrey et al. 2015). This fact is particularly important because traction forces of certain types of cells such as myofibroblasts play a key role in remodelling the matrix and modulate the activities of neighbouring cells (Layton et al. 2020). Therefore, any perturbation may lead to a vicious circle (Karnik 2003; Wang and Lin 2007), leading to unstable situations where the aortic wall never recovers stress homeostasis (Thyberg et al. 1997; Mao et al. 2015).

However, dysfunctions in the mechanical behaviour of SMCs have never been quantified in ATAAs. Only one study to our best knowledge characterized the impairment of SMC contractility in abdominal aortic aneurysms using Electric Cell-substrate Impedance Sensing (ECIS). This study was achieved for a significant cell population, but traction forces were not characterized at the single cell level (Bogunovic et al. 2019). Characterizations at the single cell level were sometimes achieved on aortic SMCs, but never on primary human SMCs. Recently, (Sugita et al. 2019) introduced a photoelasticity-based method using retardation, which is related to the difference between the first and second principal stresses and their orientation, to evaluate cellular contractile force. They showed that SMCs of low passage, which are more contractile, develop higher traction forces than SMCs of high passage. This also completes previous results obtained by (Ye et al. 2014) who carried out Traction Force Microscopy (TFM) experiments on engineered SMCs and showed that elongated SMCs have smaller traction forces, but they facilitate tone modulation by increasing its dynamic contractile range.

Accordingly, there is a still pressing need to quantify the mechanobiological changes occurring in ATAAs at single cell level with primary human cells. To address this need, we developed an in vitro technique, based on Traction Force Microscopy (TFM), to quantify SMC basal tone and compare primary SMCs from aortic aneurysms and healthy aortas. After presenting the approach which was initially tested on a commercial cell lineage (Petit et al. 2019a), we report results on three primary healthy (AoPrim) human SMC lineages and three primary aneurysmal (AnevPrim) human SMC lineages, obtained from age and gender matched donors. Eventually, we propose interpretations of the different mechanical responses of AoPrim and AnevPrim using morphological analyses and the motor-clutch model.

## Materials and methods

### Healthy and aneurysmal cell lineages

Human aortic samples were harvested on 3 deceased organ donors (with the authorization of the French Biomedicine Agency—PFS 09–007—and in accordance with the declaration of Helsinki) and, after informed consent, on 3 patients undergoing ATAA surgical repair according to protocols approved by the CHU-SE ethics committee (Centre Hospital-Universitaire ids—Saint-Etienne, France). After macroscopic examination, the 3 aortas of deceased organ donors were classified into healthy aortas according to Stary classification (Stary et al. 1994) and the Virmani list (Bentzon Jacob Fog et al. 2014).

Just after harvest (within 2 h), aortic samples were stored in physiological serum and put into the incubator at 37 °C. The extraction of SMCs was performed immediately after, by cutting the aortic tube according to its length and transferring the plane sample into a Phosphate Buffer Saline (PBS) bath. With tweezers, the adventitia was gently removed in order to remove fibroblasts, as they may grow faster than SMCs and contaminate the culture. Then, we removed the intima, in order to have only the media remaining in the Petri dish. We cut the media into small pieces and immersed them in tubes containing both elastase (Elastase, Lyophilized ESL, Worthington) and collagenase (Collagenase, Type I, powder, Gibco^™^) in PBS. The tubes were heated to 37 °C and shaken slowly during 3 h until the final solution looked cloudy (Didelot 2018).

In parallel, we coated the culture flask with fibronectin (Fn), using 10% Human Fn (Human Fn, Promocell) in PBS: we let the solution 3 h at room temperature or 30 min at the incubator before removing it from the flask. This coating was necessary for enhancing cell adhesion for aortic SMCs from primary culture. Then, we filtered the solution successively into 70 µm and 40 µm strainers in order to eliminate the remaining ECM components and keep only the SMCs. We carefully rinsed the tube that contained the solution to filter three times with 10 mL PBS. After each filtration, we centrifuged the tube that contained the filtered solution during 5 min at 1500 rpm, we eliminated the supernatant, and we suspended again the pellet with 10 mL PBS for the first time, and in 5 mL growth medium (SmGM-2, Lonza) at last. This medium promotes proliferation thanks to high (5%) fraction of Fetal Bovine Serum (FBS) and other specific components including growth factors.

Finally, we transferred the cell suspension into the flask and completed with 5 mL of growth medium. The flask was put into the incubator at 37 °C 5% CO_2_ for 2 weeks for sufficient cell growth, during two passages. At the end of this initial step of primary culture, we froze the SMCs at passage 2 into 1.5 mL aliquots containing a freezing solution composed of 80% complete medium, 10% SVF, and 10% DMSO and stored them into liquid nitrogen. Each aliquot contained between 2 and 6 million SMCs.

Aliquots of cells coming from healthy donors, named AoPrim onwards, were extracted according to the same protocol and stored in a biobank (INSERM U1148, Bichat Hospital, Paris, France) and shipped to our laboratory for the purpose of this study. Aliquots of cells coming from ATAA patients, named AnevPrim onwards, were stored in similar conditions directly in our laboratory (INSERM U1059, Saint-Etienne, France). Characteristics of the donors are reported in Table 1. In order to minimize the effects of gender and age, all donors were of the same age range (65–79 years old).

**Table 1:** Characteristics (Gender, age) of the donors for the 3 healthy SMC lineage and for the 3 aneurysmal SMC lineage. The aortic diameter, the type of aortic valve and the presence of hypertension is also reported for the 3 donors harboring an ATAA

SMCs lineage	Gender, age	Aortic diameter (mm)	Aortic valve	Hypertension
AoPrim1	H, 74	< 35	Tricuspid	N/A
AoPrim2	H, 65	< 35	Tricuspid	N/A
AoPrim3	F, 69	< 35	Tricuspid	N/A
AnevPrim1	H, 72	57	Tricuspid	Yes
AnevPrim2	H, 69	48	Tricuspid	No
AnevPrim3	F, 79	56	Mechanical prosthesis for 34 years	Yes

### Cell culture

After thawing, the cells were transferred into a T-75 flask for an entire week in growth medium (SmGM-2, Lonza). The cells were incubated at 37 °C and 5% CO_2_ to maintain the pH at 7.2–7.4. Then, SMCs were cultured one week more in a basal medium (SmBM, Lonza), without FBS and containing 0.04% heparin for differentiation. Once they reached 50–70% confluence, we used a standard cell detachment protocol using a trypsin treatment with a low trypsin—EDTA solution (0.025% Trypsin and 0.75 mM EDTA (1X), Sigma) to break down the focal adhesions in the culture dish without damaging the cells. Then, the cells in suspension were used for subculturing or for sample preparation.

### Sample preparation

**Fig.1:**
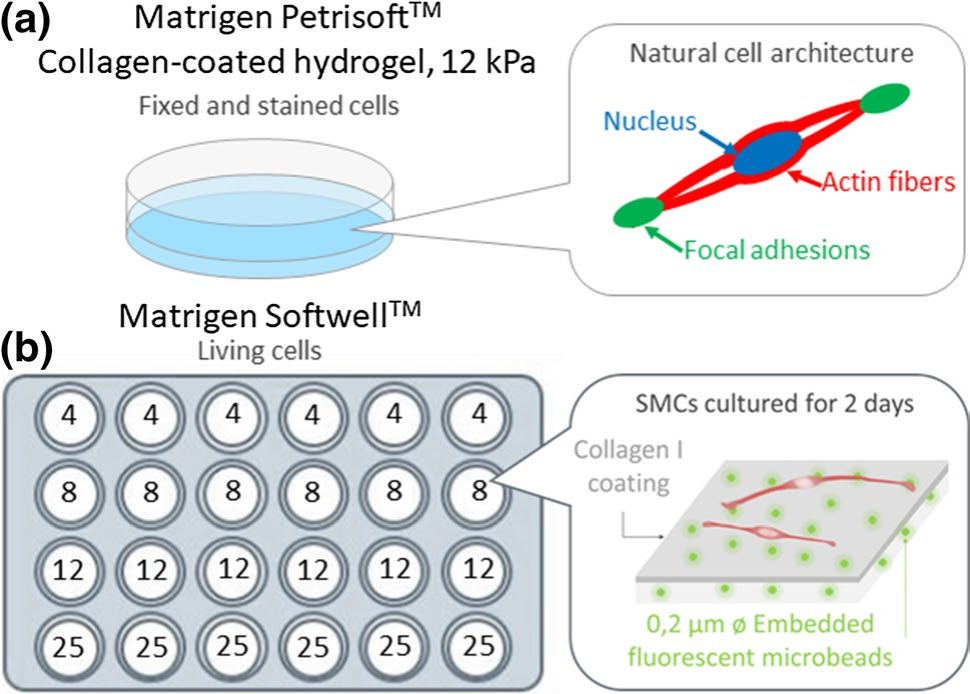
**a** Collagen-coated 12 kPa hydrogel in a Petri dish containing adhering SMCs and schematic of a single SMC adopting the natural spindle shape. **b** The Matrigen 24-well plate contained 4 lines of 6 hydrogel samples with the same stiffness (4, 8, 12, and 25 kPa). The gels were previously covered by a collagen I adhesive pre-coating and included yellow-green 0.2 µm diameter fluorescent microbeads

Previously starved SMCs (see subsection 2.2) at passage 3 were transferred in Petri dishes (Fig. 1a) or 24-well plates (Fig. 1b) containing ready-to-use hydrogels with different stiffness properties (Cell Guidance System: Petrisoft^™^ 35, collagen pre-coated and Softwell^™^ 24, collagen pre-coated, Softrack 0.2 µm diameter microbeads labelled with yellow-green fluorescent dye, customed stiffness values). These hydrogels were made of a 400-µm-thick layer of polyacrylamide, which was assumed to be linear elastic within the range of strains considered in our study, and to have the same behaviour in both tension and compression. The gel plates were 12 mm diameter, which is sufficiently large with respect to the cell size (about 0.4 mm long). Moreover, the collagen I coating provided a physiological surface for cell adhesion and culture. Since the coating is a protein monolayer, whereas the hydrogels are 400 µm thick, we assumed that the coating only affects marginally the mechanical behaviour of the substrate.

According to previous observations with gels from 0.5 to 50 kPa (Petit et al. 2019a), we used 24-well plates of intermediate stiffness values: 4, 8, 12, and 25 kPa, as these stiffness values allowed reproducible quantification of bead displacements whilst lying within a range compatible with soft tissue stiffness. The stiffness values indicated by the supplier were verified using AFM indentation before performing the experiments.

About 50 000 cells per Petri dish and 20 000 cells per well were seeded on the hydrogels and incubated in basal medium for two days before the experiments. This duration was sufficient to ensure a good spreading of SMCs, which generally adopted a specific elongated spindle shape.

### Traction force microscopy (TFM) to measure SMC basal tone

We used a Carl Zeiss Axio Observer.Z1 station with the Zen software. The station fits an incubating chamber in which we maintained the previously cultured hydrogels at 37 °C, 5% CO_2_. The Green Fluorescent Protein (GFP) channel was used to track the displacement of the green fluorescent microbeads embedded in the gels. Then, we used the phase contrast channel to image the cells and to measure, for each of them, their morphology (Sect. 2.5). The best magnification was obtained with the Plan-Neofluar 20x/0.4 objective, which resulted in a resolution of 0.323 µm per pixel. This resolution was kept for all further image acquisition.

The method consisted in recording one frame per 30 s throughout 3–5 min total duration, until the cells detach completely from the surface after trypsin treatment. In order to obtain the trypsin-induced detachment of SMCs, the medium was first removed from the well and the cells were gently washed once with PBS, as some medium components tended to inhibit the trypsin effect. A little amount of PBS remaining on the surface of the hydrogels was sufficient to keep the cells hydrated before trypsin treatment. Then, we focused with the microscope on an area of interest around several adhering and sufficiently isolated cells. The field of view of the objective allowed the selection of 1–4 cells per well at the same time.

Normally, cells detached from the gels within 1–2 min, and a deformation was observed in the gel, with localized motions of the microbeads that was induced by the release of SMCs initial basal tone. Once the cells were detached by trypsin treatment, the well was not reusable. This process was repeated for every well of the plate successively, on at least 2 plates for each cell line. The resulting images were processed using Digital Image Correlation (DIC) first, to obtain the corresponding displacement and strain fields on all the deformed images. Then, we applied a custom Matlab^®^ code for selecting an area around each cell anchorage point and we derived the traction force value. The full theory and method related to traction force measurement are explained in a previous paper (Petit et al. 2019a).

### Cell morphology

We also performed morphological and histochemical characterizations in the same conditions as the TFM experiments (adhering on collagen-coated gels of 12 kPa stiffness).

From the phase contrast channel images obtained with the microscope (Fig. 3), the contours of isolated cells selected for TFM measurements were segmented manually and used to measure the cell length, width, orientation, and surface area (Fig. 5). For multidirectional cells, we determined the principal axis and measured the length along this axis and the width along the perpendicular direction. Moreover, we imaged supplemental AoPrim and AnevPrim SMCs (only on 12 kPa hydrogels) using fluorescent microscopy in order to characterize the distribution of filamentous actin (F-actin) and of the α-SMA actin isoform, which is known to be involved in the contractile apparatus of SMCs. More details about the fluorescent microscopy protocol are provided in the supplemental materials (Fig. S1, Appendix A).

### Statistics

For each cell population, we performed statistical analyses on the TFM measurements. We obtained traction forces on 6 wells for each stiffness value (4, 8, 12 and 25 kPa), which corresponds in total to 16–24 cells for AoPrim on each stiffness and 27–54 cells for AnevPrim on each stiffness. We used a Mann–Whitney nonparametric test in order to compare the distribution of two independent samples, with different sample size. The null hypothesis was that data from the two tested populations were from continuous distributions with equal medians. We tested populations (one per stiffness value) two by two successively and displayed the results as a boxplot using Matlab^®^. A *p*-value below 0.05 was interpreted as a significant result, namely that the null hypothesis was rejected at this significance level.

### The motor-clutch model

**Fig. 2:**
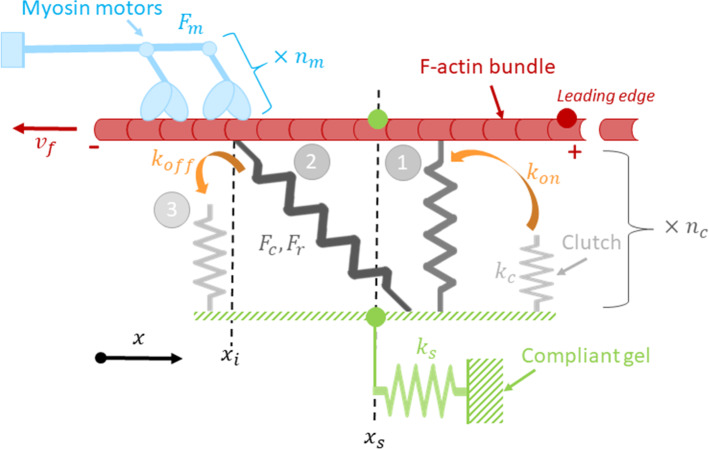
Schematic representation of the motor-clutch model. Once engaged (1), the *i*th clutch undergoes a load (2) and fail (3) cycle. This can be generalized to *n*_*m*_ myosin motors and *n*_*c*_ clutches (Chan and Odde 2008; Bangasser et al. 2013)

Firstly introduced by Chan and Odde (2008), the motor-clutch model simulates the filopodium dynamics involving the main components of the cytoskeletal structure (Bangasser et al. 2013; Bangasser and Odde 2013). In this model, it is assumed that myosin molecular motors drag actin bundles, resulting in filament sliding. Cell traction forces are then transmitted to the compliant substrate through the focal adhesions, which consist in a certain number of molecular clutches. A clutch is represented by a single spring which can fail following a probabilistic law. A schematic of the model is represented in Fig. 2.

While the clutch is still engaged on the substrate, the cytoskeletal tension keeps increasing until failure. Consequently, each clutch undergoes successive cycles of loading/failure, but the behaviour depends on the stiffness of the substrate. Therefore, the model relies on the probability that a single considered clutch is bound or unbound at a given time. The motor-clutch Matlab code available online (Odde 2017) simulates binding and unbinding events of a fixed duration using the Monte Carlo method. The temporal change in the probability of binding for a single clutch is given by,

1 $$ \frac{{dp_{b,i} }}{dt} = \left( {1 - p_{b,i} } \right) \cdot k_{on} - p_{b,i} \cdot k_{off,i}^{*} $$

where $p_{b,i}$ is the probability that the *i*th clutch is bound, and $k_{on}$ is the binding rate. The dissociation rate $k_{off,i}^{*}$ for a single clutch follows an exponential law as in the Bell model (Bell 1978),

2 $$ k_{off,i}^{*} = k_{off} .e^{{{\raise0.7ex\hbox{${F_{c,i} }$} \!\mathord{\left/ {\vphantom {{F_{c,i} } {F_{r} }}}\right.\kern-\nulldelimiterspace} \!\lower0.7ex\hbox{${F_{r} }$}}}} $$

where $k_{off}$ is the unbinding rate of the unloaded clutch, $F_{c,i}$ is the tension undergone by the clutch, and $F_{r}$ is the rupture force of the bond. According to the assumption of a single linear elastic spring, $F_{c,i}$ is linked to the spring constant $k_{c}$ and the spring extension $\Delta L$ such as,

3 $$ F_{c,i} = k_{c} \cdot \Delta L = k_{c} \cdot \left( {x_{i} - x_{s} } \right) $$

where $\Delta L$ is the difference between the position of the actin-clutch interface $x_{i}$ and the position of the substrate-clutch interface $x_{s}$. At the anchorage point of the cell that contains a total number of clutches $n_{c}$, we can express the equilibrium such as,

4 $$ F_{c} + F_{s} = 0 $$

where $F_{c}$ is the total force exerted by $n_{c,eng}$ engaged clutches and $F_{s}$ the reaction force of the substrate at the anchorage point. Equation 4 can be explicitly rewritten as,

5 $$ k_{s} \cdot x_{s} - k_{c} \mathop \sum \limits_{i = 1}^{{n_{c,eng} }} \left( {x_{i} - x_{s} } \right) = 0 $$

The position of the substrate may be eventually expressed as,

6 $$ x_{s} = \frac{{k_{c} \cdot \mathop \sum \nolimits_{i = 1}^{{n_{c,eng} }} x_{i} }}{{k_{s} + n_{c,eng} \cdot k_{c} }} $$

The $x_{i}$ position is updated for each event according to,

7 $$ x_{i} \mapsto x_{i} + v_{u} \cdot \left( {1 - \frac{{k_{s} \cdot x_{s} }}{{n_{m} \cdot F_{m} }}} \right)*dt $$

As the substrate, spring constant $k_{s}$ is involved in Eq. 7, and the cytoskeleton dynamics is sensitive to the substrate stiffness. Nevertheless, this sensitivity may be lost if the total myosin motor stall force is reached. This force is the number of motors $n_{m}$ times the stall force of a single motor $F_{m}$.

Based on these equations, 10^5^ events can be simulated with the Matlab code in successive iterations updated from a fixed duration of 0.005 s, until reaching a steady state. At the beginning of each step, molecular clutches are first allowed to bind randomly with a binding rate $k_{on}$ (Eq. 1). Then, random disengagement is modelled at an unbinding rate $k_{off}^{*}$, while the loading is increased (Eq. 2). A random network of both bound and unbound springs is obtained. The final position of the substrate $x_{s}$, is updated at every event with the new number of engaged clutches $n_{c,eng}$ (Eq. 6). Then, the net force at the anchorage point is zero if $F_{c} = F_{s}$.

Otherwise, $F_{c} = k_{s} \cdot x_{s}$ (Eq. 4 and Eq. 5).

Knowing $k_{s}$ and $x_{s}$, $F_{c}$ is calculated for each event, and the mean force value can be estimated for each substrate stiffness.

Typical parameter values for the motor clutch model were reported by (Bangasser et al. 2013). However, the motor-clutch model with these parameter values did not match accurately our TFM measurements. Therefore, we had to calibrate four of these parameters ($n_{m}$, $k_{on}$, $n_{c}$, $k_{off}$) in order to adjust the model to our experimental results.

### Fitting of the motor-clutch model with experimental data

We performed a systematic search using an optimization algorithm in Matlab. We used the initial Matlab code available online (Odde 2017) for the motor-clutch model for launching several successive simulations with progressive changes in the parameters we considered as variable, namely $n_{m}$, $k_{on}$, $n_{c}$, $k_{off}$. Then, we used the data from these simulations in order to compare it with the experimental median traction force values measured on each gel stiffness. In this way, we defined an objective function as the sum of squares of differences between predicted traction force values from the model and the median of experimental values.

The first iteration consisted in the adjustment of the $k_{on}$ and $k_{off}$ rates, in order to fit first the whole range of theoretical and experimental traction force values for all cell lineages. Then, the same $k_{on}$ and $k_{off}$ rates were kept for all the cell lineages. We eventually adjusted the $n_{m}$ and $n_{c}$ parameters. We repeated the method until the model was sufficiently close to our experimental values (the stopping criterion was a variation of less than 1% between two successive iterations).

## Results

### Measurement of cell traction forces

TFM experiments were carried out on three primary healthy (AoPrim) human SMC lineages and three primary aneurysmal (AnevPrim) human SMC lineages, adhering to collagen-coated hydrogels with 4 different stiffness values. In total, we measured traction forces of 759 primary human aortic SMCs which were sorted in 24 populations (6 cell lineages × 4 substrate stiffness). In order to automatize the process, 24-well plates containing 6 wells of each stiffness values (4, 8, 12, 25 kPa) were used for each cell lineage. For each well, we measured the traction forces for about 1 to 4 cells depending on their location. For each stiffness value, we could finally evaluate the traction forces for a number of cells varying between 16 and 24 for AoPrim and 27–54 for AnevPrim lineages.

**Fig. 3:**
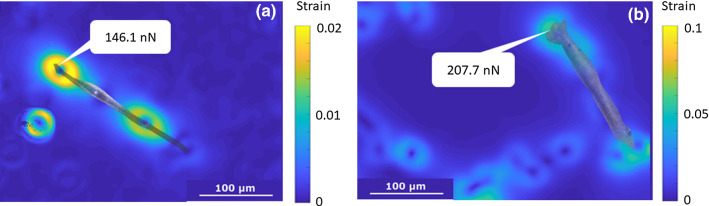
Morphologies of a healthy SMC from AoPrim3 lineage (**a**) and of an aneurysmal SMC from AnevPrim3 (**b**) obtained from the phase contrast channel of the microscope and superimposed onto the strain maps measured for TFM analyses. The yellow-blue double pattern corresponds to the local traction force exerted by the cell on the compliant hydrogel. The force derived from the TFM analysis is also displayed for each cell. (Color figure online)

Examples of 2 primary SMCs (one AoPrim and one AnevPrim) among the 759 traction force measurements are shown in Fig. 3. These SMCs were artificially detached from the collagen by a trypsin treatment, which removed the traction forces they exerted on the gel initially, corresponding to the cell basal tone. These forces were localized on the ending part of the cell and oriented from each pole towards its nucleus. At it can be seen in Fig. 3, cells had developed anchorage points on the substrate, where the strain maps exhibit a traction-compression pattern. This local pattern was related to the traction force as explained in (Petit et al. 2019a).

**Fig. 4:**
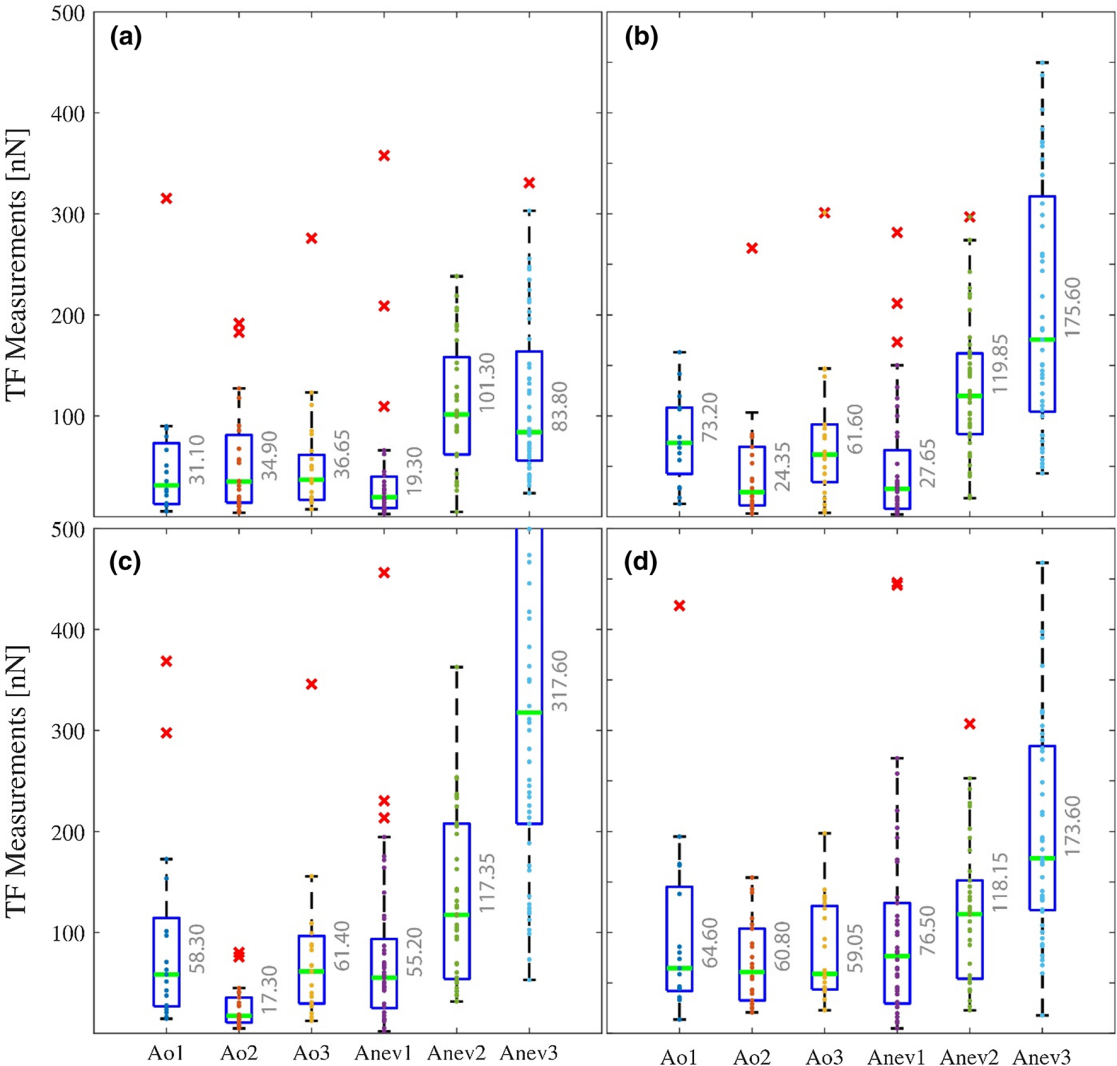
Boxplots showing the distribution of the measured traction forces (TF) (experimental data) for AoPrimX and AnevPrimX lineages for each gel stiffness: 4 kPa **a**, 8 kPa **b**, 12 kPa **c**, and 25 kPa **d**. Median values are reported for a clearer comparison. Significance between two populations was found using a Mann–Whitney test. Significance was stated there for *p* < 5% (results reported in supplemental materials, appendix B, Tab. S3)

For each gel stiffness, the obtained traction force values were plotted in Fig. 4 as boxplots for each of the six cell lineages (3 AoPrim on the left hand side and 3 AnevPrim on the right hand side). The traction forces are globally heterogeneous. They were also represented as histograms in the supplemental materials (Appendix B—Figs S3, S4 and S5) for each of the 24 populations, showing that they exhibit generally an exponential distribution, which is still consistent with our previous observations on a commercial cell line of aortic SMCs (Petit et al. 2019a).

The median traction force for each group is also reported in Fig. 4. The median seemed more relevant than the mean given the large variability, but mean values are also reported in supplemental materials (Appendix B, Tab. S1). We observed inter-individual variations between the median traction forces of each group of cells. We found that the three AoPrim had similar median values, excluding the values of AoPrim2 at 8 kPa and 12 kPa. The trend is a median traction force of about 30 nN on gels of 4 kPa and a median traction force between 60 and 70 nN on gels of 8 kPa, 12 kPa and 25 kPa (again excluding the values of AoPrim2 at 8 kPa and 12 kPa). The effect of substrate stiffness on traction forces is highlighted in the supplemental materials (Appendix B, Figs. S6 and S7). Globally, it appears that median traction forces would increase when the substrate stiffness increases up to 8 kPa or 12 kPa and then remain constant or even decrease for larger substrate stiffness.

Although the median traction forces of AoPrim remained in the 30–70 nN, the AnevPrim showed a dramatically larger range with values that were significantly larger, especially for AnevPrim2 and AnevPrim3, which showed medians beyond 100 nN and even a median reaching 300 nN for AnevPrim2 on the 12 kPa substrate. Interestingly, all AnevPrim showed increase in the traction forces between the softest substrates and the other substrates. Moreover, AnevPrim2 and AnevPrim3 both showed maximal median values on the 12 kPa substrate, which is similar to previous results obtained on a commercial aortic SMC lineage (Petit et al. 2019a).

We did not indicate the statistical significance of differences between each group in Fig. 4 for the sake of readability of the figure. However, the p-values for the comparison of each group are reported in the supplemental materials (Appendix B, Tab. S3). It is shown that AnevPrim2 and AnevPrim3 are always significantly different to the other cell lineages, with larger traction force values.

Cross comparison between the different substrate stiffness is also shown as boxplots in the supplemental materials (Appendix B, Figs S6 and S7). This shows that all the cell lineages exhibit statistically significant differences in the traction forces they apply on gels of different stiffness, except AnevPrim2 which showed median traction forces always between 100 and 120 nN.

Due to the large variability of traction forces, we also looked at global trends such as the fraction of low (< 60 nN) and high (> 200 nN) traction forces. Results are reported in supplemental materials (Appendix B, Tab. S2). About 50% of the AoPrim1 applied traction forces in the range 0–60 nN, whereas less than 20% of the AnevPrim applied traction forces in this range.

### Measurement of SMC morphology and architecture

**Fig. 5:**
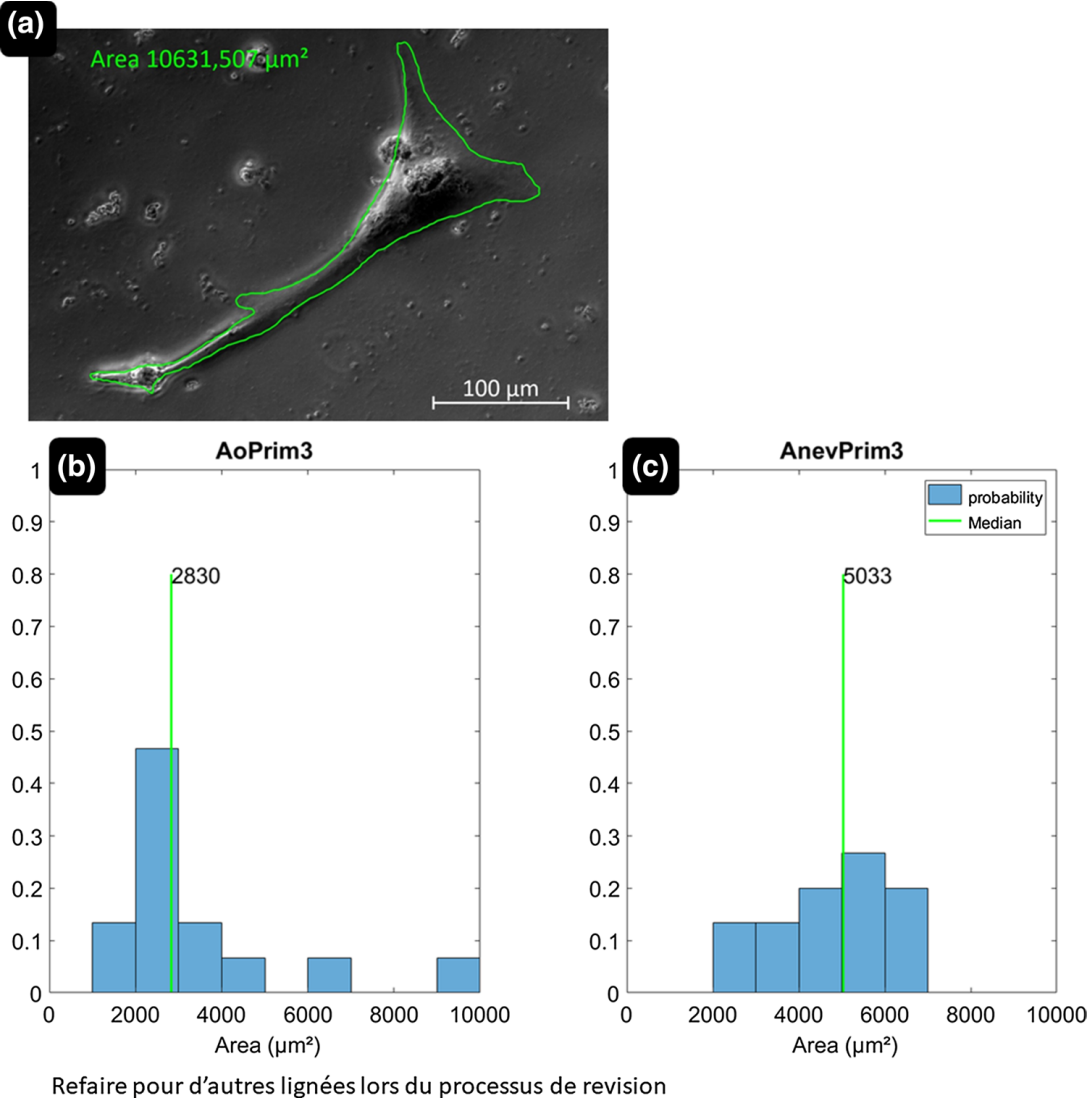
Image of a SMC obtained with the phase contrast channel of the microscope. The contour of the cell was segmented manually, and the surface area of each cell was assessed with the image processing software. The distribution of surface areas for AoPrim SMCs and AnevPrim SMCs is showed as histograms in (**b**) and (**c**), respectively. The surface area of AnevPrim SMCs is significantly larger than the one of AoPrim SMCs. Statistical significance between the two populations was found using a Mann–Whitney test for *p* < 5% (*p* = 0.0279). (Color figure online)

As the results seem to indicate a larger fraction of stronger SMCs in the AnevPrim populations than in AoPrim populations, we also compared their respective morphology and composition. Most of AoPrim naturally showed a pronounced uniaxial spindle-shape with lower surface area compared to AnevPrim SMCs. The morphology of AnevPrim SMCs appeared more often multipolar, with several other directions of spreading and larger membrane extensions. The distribution of cellular areas are reported in Fig. 5, showing a trend towards bigger cells for AnevPrim, which appeared more spread, and with less prominent membrane extensions.

Fluorescent imaging of cells from both cell lineages showed that the SMC cytoskeleton was particularly dense around the cell, by forming long stress fibres on each side. For AnevPrim, in which the membrane extensions were larger, we clearly noticed an organization of criss-crossed actin fibres that formed the lamellipodium. Conversely, in lateral stress fibres, the actin fibres were closer and more parallel to each other. F-actin is more concentrated into long thin fibres all along the cell length for long and thin AoPrim (Appendix A, Fig. S1). In both cases, the F-Actin was dispersed into the whole cell volume and defined the cell shape.

In addition, the α-SMA specific isoform of SMCs was concentrated around the nucleus and along the boundaries of the cells. The quantification of the fluorescent intensity was performed on both α-SMA and F-actin corresponding channels for *N* = 22 cells from each lineage (Appendix A, Fig. S2). A significant increase in α-SMA expression was showed for AnevPrim cells (Appendix A, Fig. S3a). Nevertheless, we did not notice any significant difference for F-actin (Appendix A, Fig. S2b).

### Motor-clutch model

**Fig. 6:**
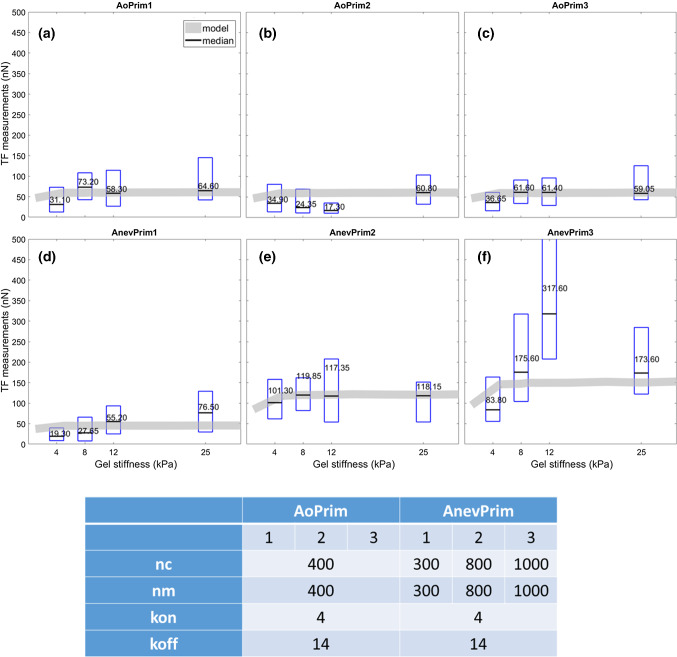
Motor-clutch simulations of traction forces obtained with optimal parameter sets are compared to the measured traction forces. AoPrim and AnevPrim SMCs had significantly smaller traction forces on the 4 kPa gels than on gels of larger stiffness, which was also predicted by the model. For each substrate stiffness, the mean traction forces measured experimentally are plotted with a red star symbol. Results for AoPrim1 (**a**), AoPrim2 (**b**), AoPrim3 (**c**), AnevPrim1 (**d**), AnevPrim2 (**e**), and AnevPrim3 (**f**)

Finally, we modelled the median of each cell lineage with the motor-clutch model to reproduce the stiffness effect shown by the TFM results. Except for AoPrim2, the model could predict forces in good agreement with the experimental TFM results after tuning the model parameters (*n*_*m*_, *n*_*c*_, *k*_*on*_, and *k*_*off*_). Results are shown in Fig. 6, and the model parameters are reported in Table 2. A larger number of motors and clutches had to be used for AnevPrim2 and AnevPrim3 given the significantly larger traction forces in these populations.

**Table 2:** Motor-clutch parameters with the values identified for the six cell lineages

Parameter	Symbol	AoPrim			AnevPrim		
Cell lineage		1	2	3	1	2	3
Number of motors	$n_{m}$	400	400	400	300	800	1000
Motor stall force	$F_{m} \left( {nN} \right)$	2			2		
Unloaded actin velocity	$v_{u} \left( {nm/s} \right)$	120			120		
Number of clutches	$n_{c}$	400	400	400	300	800	1000
Rupture force	$F_{r} \left( {nN} \right)$	2			2		
Binding rate	$k_{on} \left( {{\text{s}}^{ - 1} } \right)$	4	4	4	4	4	4
Unloaded unbinding rate	$k_{off} { }\left( {{\text{s}}^{ - 1} } \right)$	14	14	14	14	14	14
Spring constant	$k_{c} \left( {{\text{N}}/{\text{m}}} \right)$	0.8			0.8		

## Discussion

### Differences between healthy and aneurysmal SMCs

In this study, we achieved for the first time single-cell TFM experiments on primary human aortic SMC lineages coming from age and gender matched healthy and aneurysmal donors and found that a significant fraction of aneurysmal SMCs apply traction forces larger than 100 nN, whereas the traction forces of healthy SMCs generally lie in a 30–70 nN range.

Characterization of the traction forces at the single cell level on primary human aortic SMCs was challenging, but it permitted to assess for the first time the basal tone of isolated SMCs (759 in total), avoiding to average the response of a cell population with possibly different phenotypes (Liu and Gomez 2019). For instance, (Bogunovic et al. 2019) characterized the impairment of SMC contractility in abdominal aortic aneurysms but not at the single-cell level.

TFM characterizations at the single cell level were sometimes achieved on aortic SMCs, but never on primary human SMCs (Sugita et al. 2019). It was shown for instance by (Ye et al. 2014) that elongated engineered SMCs have smaller traction forces, but they facilitate tone modulation by increasing its dynamic contractile range.

It was shown by (Sugita et al. 2019) that SMCs of low passage (3) are more contractile and develop higher traction forces than SMCs of high passage (12). Our experimental conditions ensured the use of cells at passage 3, which are known to have significant contractility as shown by Murray et al. (Murray et al. 1990). Moreover, serum deprivation was used to maintain significant contractility. The very good preservation of AoPrim/AnevPrim contractile behaviour is also indicated by clear spindle-shaped SMCs and high expression of α-SMA (Rzucidlo et al. 2007; Timraz et al. 2015) (data in Supplemental materials, Appendix A). These filaments are well known for their high contractility and are present only in cell types that need to contract strongly in the human body, like myofibroblasts and SMCs (Anderson et al. 2004; Goffin et al. 2006; Chen et al. 2007).

Morphological analyses revealed that healthy SMCs tend to be longer than aneurysmal SMCs, which showed thicker morphologies (example shown in Fig. 3). Substantially fragmented and disorganized F-actin fibres were also previously reported (Riches et al. 2018).

Moreover, AnevPrim SMCs showed more frequently a multipolar shape. Such SMCs may be more prone to migrate as reported for synthetic SMCs (Thyberg et al. 1990; Humphrey 2002). In addition, their less elongated and thicker shape (significantly larger areas shown in Fig. 5) would indicate a hypertrophic morphology, characteristic of the synthetic phenotype (Timraz et al. 2015) in which synthetic organelles need to be more developed (Owens et al. 1981; Humphrey 2002). The morphological differences observed in AnevPrim2 and AnevPrim3, along with the related large traction forces, seem to indicate remodelling of the cytoskeleton of a large number of these cells. However, not all AnevPrim SMCs undergo such remodelling. For instance, the median of traction forces in AnevPrim1 is close to the one of AoPrim cells. This may be accounted for by inter-individual differences. Although all the donors had a tricuspid aortic valve (Tab. 1), both AnevPrim2 and AnevPrim3 donors had aortic valve dysfunctions (AnevPrim3 donor harbored a mechanical prosthesis) inducing significant hemodynamic disturbance, which are traditionally typical of bicuspid aortic valves, and which were shown to induce significant remodelling in the aortic wall (Guzzardi et al. 2015; Michel et al. 2018; Jayendiran et al. 2020).

### Relation between traction forces and cellular function

The basal tone of aortic SMCs is an essential aspect of the arterial function (Petit et al. 2019a, b). In the current study, we show that aneurysmal SMCs tend to have a different mechanical response, which is consistent with the quantitative study of (Bogunovic et al. 2019) who showed impaired maximum contraction on populations of SMCs of 23% abdominal aortic aneurysm patients (5 out of 21).

Other previous studies reported several differences for aneurysmal SMCs relatively to healthy ones in terms of morphology (larger shape, granularity around nucleus…) and expression (cytoskeletal organization, senescence markers…) (Qiu et al. 2010; Riches et al. 2018). They suggested that aneurysmal SMCs show some signs of senescence (Riches et al. 2018). Moreover, previous work about senescent SMCs revealed higher stiffness and lower contractility (Qiu et al. 2010; Lacolley et al. 2018). The reduced contractility of AnevPrim SMCs would imply a reduction in their responsiveness to vasoactive agents, but also a possible increase in their basal traction forces, as shown by (Ye et al. 2014). Therefore, large traction forces in our experiments could be partially explained by the senescent-like behaviour of aneurysmal SMCs. Another explanation may be that some AnevPrim cells behave as myofibroblasts, which modulate their traction forces in function of their synthetic behaviour (Layton et al. 2020). However, this should be confirmed by further investigations, as cellular stiffness and contractility depend not only on the degree of differentiation but also on the general molecular environment of vSMCs (Lacolley et al. 2018).

We assessed the influence of the cell shape on the contractility, and we found no evident correlation between traction force values and cell length. SMCs do not seem to increase their tension when they become longer. Their area seems to be more representative of their strength, as shown in Fig. 5.

Since synthetic SMCs express more migration and proliferation (Thyberg et al. 1990; Humphrey 2002), their cytoskeleton may undergo quicker turnover to adapt to cell deformation and movement, with different amount of myosin motors, shorter bond lifetimes and eventually quicker actin flow. Therefore, we tested the motor-clutch model for simulating our traction force measurements and noticed that a larger number of motors and clutches were required to model the response of the AnevPrim SMCs (Fig. 6). The motor-clutch model was motivated by the effects of the substrate stiffness on the traction forces. The obtained results showed clearly that both AoPrim and AnevPrim SMCs had significantly smaller traction forces on the 4 kPa gels than on gels of larger stiffness, which was also predicted by the model (Fig. 6). Despite these interesting results, translating the model parameters into physical observations is still a work in progress. Further analyses should look at protein expressions in order to find correlations between parameter values in the motor clutch model and levels of protein expression such as integrins and myosin.

Under stress free conditions, aortic walls have normally a stiffness around 40 kPa and can reach until 110 kPa in atherosclerosis (Sazonova et al. 2015). In pressurized arteries, the stiffness can even reach several MPa. Therefore, our experimental conditions on gels between 4 and 25 kPa are significantly lower than real tissue. However, in the specific structure of the medial layer, SMCs are arranged into lamellar units and connected to the elastic laminae by bundles of elastic microfibrils (Clark and Glagov 1985; Davis 1993; Humphrey et al. 2015), which may act as compliant springs between stiffer material and shield the stress. Therefore, SMCs may sense only an apparent stiffness due to this specific organization that should be lower than 40 kPa. Moreover, local mechanics are dominated by fibre bending, whereas the gels are dominated by a stretching-dominated regime. Moreover, the actual in vivo stiffness of aortic tissue values in the macroscopic stretching-dominated regime does not inform about local micromechanics sensed by the cell. In contrast, experimentally imaged connective tissue architectures were found to yield a strikingly broad range of local stiffness, spanning roughly two decades (Beroz et al. 2017). Therefore, SMCs must adjust their size, shape, and position to adapt to the multiple stiffness values in the tissue. An impaired mechanosensitivity and mechanotransduction could explain the different adaptation of aneurysmal SMCs to a similar environment (Layton et al. 2020). This may suggests that aortic disease results in cell reprogramming (Michel et al. 2018). But it is still unclear if they are only expressed through phenotypic switching or if deeper changes occur as well (Gomez et al. 2015). Moreover, the high inter-cellular variation shown by our results suggests cell reprogramming does not occur uniformly and may depend on other external factors.

### Limitations and future work

One of the main limitations of this study is that in vivo microenvironment was only mimicked in 2D by the choice of the adhesive coating, but not fully reproduced due to its biochemical and biophysical complexity in 3D. In the aorta, fibrillar type I collagen is naturally present in the media, and SMCs tend to align along collagen fibrils (Wagenseil and Mecham 2009) but also with the direction of maximum principal stress (Fujiwara and Uehara 1992; Humphrey 2002; Wagenseil and Mecham 2009). Accordingly, they arrange predominantly with a circumferential orientation, permitting the regulation of stress and strain in this direction (Clark and Glagov 1985; Hayashi et al. 2012). Choosing type I collagen coating for TFM experiments seemed relevant, even though the difficulty to extrapolate in vivo conditions from an in vitro uniform 2D environment remains the first limitation of this study.

Another limitation is related to the large variability of the results. Even if it is the first time ever that traction forces are reported in the literature on more than 750 primary human aortic SMCs from healthy and aneurysmal patients, more than tenfold datasets would be needed to understand the different effects related to the regulation of traction forces in aortic SMCs. This is also related to the diversity of ATAA etiologies (Michel et al. 2018). Although the 3 ATAA donors were from the same age range, there showed diversity in some of their characteristics as reported in Table 1. For instance, one of them had a mechanical aortic valve and two of them were diagnosed hypertension. None of them presented a genetic disease related to aortopathies. Another source of variability may be the location of the initial aortic sample. Whereas all samples came from the mid-ascending aorta, AnevPrim1 also contained the sino-tubular junction and a fragment of the aortic root, which may have induced differences between AnevPrim1 and the two other AnevPrim lineages.

In addition, the morphological analyses and the α-SMA/F-actin expressions reported in supplemental materials should be completed with some other markers such as smoothelin, myosin, or calponin, in order to understand better the cytoskeletal changes of SMCs. Since we suggest that a larger fraction of SMCs express a synthetic rather than contractile phenotype in ATAAs, we should also test their responsiveness to agonists. For instance, (Ye et al. 2014) carried out Traction Force Microscopy (TFM) experiments on engineered SMCs and showed that elongated SMCs have smaller traction forces, but they facilitate tone modulation by increasing its dynamic contractile range. Similar investigations will be varied out on primary human aortic SMCs in the future. Future work should also look closer at focal adhesions. SMC adhesion could be assessed while staining the focal adhesions and measure their surface in order to express the force per surface unit. The use of deformable micropatterns may also be a good alternative (Win et al. 2017).

## Conclusion

In summary, we found that the basal traction forces applied by representative populations of human primary aortic smooth muscle cells, when cultured onto compliant hydrogels of different stiffness (4, 8, 12, 25 kPa), exhibit large heterogeneity even for cells coming from the same tissue. Despite these heterogeneities, we observed that: 1. the traction forces were significantly larger on substrates of stiffness larger than 8 kPa; 2. traction forces in SMCs from ATAAs were significantly higher than in SMCs from healthy aortas. We modelled computationally the dynamic force generation process in SMCs using the motor-clutch model and found that it accounts well for the stiffness dependent traction forces. We conclude that phenotype changes occurring in ATAAs, which were previously known to reduce the expression of elongated and contractile SMCs (rendering SMCs less responsive to vasoactive agents), tend also to induce stronger SMCs. Future work aims at understanding the causes of this alteration process in aortic aneurysms.

## Electronic supplementary material

Below is the link to the electronic supplementary material.

Supplementary material 1 (PDF 1231 kb)
