# Supplementary material for: Regulation of SMC traction forces in human aortic thoracic aneurysms
Source: Biomech Model Mechanobiol. 2021 Jan 15;20(2):717–31. doi: 10.1007/s10237-020-01412-6 (PMC7979631; doi:10.1007/s10237-020-01412-6)
Supplement: Supplementary file 1 — Supplementary material 1 (PDF 1231 kb) [file 10237_2020_1412_MOESM1_ESM.pdf]

Supplemental Materials

Regulation of SMC Traction Forces in Human Aortic Thoracic Aneurysms.

Claudie Petit[1], Ali-Akbar Karkhaneh Yousefi[1], Olfa Ben Moussa[1], Jean-Baptiste Michel[3], Alain Guignandon[2], Stéphane Avril [1]

[1] Mines Saint-Etienne, Université de Lyon, INSERM, U 1059 SAINBIOSE, F - 42023 Saint-Etienne France.

[2] Université Jean Monnet, Université de Lyon, INSERM, U 1059 SAINBIOSE, F - 42023 Saint-Etienne France.

[3] UMR 1148, Laboratory for Translational Vascular Science, Inserm and Paris 7- Denis Diderot University, Xavier Bichat Hospital, 75018 Paris, France

Corresponding Author:

Stéphane Avril
E-mail: avril@emse.fr

ORCID numbers:

Claudie Petit: 0000-0003-3817-8456
Alain Guignandon: 0000-0002-0745-5417
Stéphane Avril: 0000-0002-8604-7736

TABLE OF CONTENT

Appendix A. SMC architecture and morphology.

Fig. S1 Examples of morphological and fluorescent intensity measurements on several SMCs. Three images of healthy SMCs are shown in (a) and three images of aneurysmal SMCs are shown in (b). The red channel is for F-actin and the green channel for α-SMA. The intensity values were coded on 12 bits (i.e. 0 for black and 4096 for white). The fluorescent images were post-processed manually (segmentation of their contours) with the Zen Software®.

Fig. S2 Distributions of mean intensity values for α-SMA (GFP channel) (a) and F-Actin (Ds-RED channel) (b). Median values are reported for a clearer comparison. A significant difference was found between α-SMA values of healthy and aneurysmal cells using the Mann-Whitney statistical test.

Appendix B. Full TFM data on 24-well plates.

Tab. S1 Mean traction force values of the experimental data for AoPrim and AnevPrim SMCs on every gel stiffness. ΔF is the difference between maximal and minimal values on the whole stiffness range.

Tab. S2 Fraction of traction force values below 60 nN and above 200 nN for AoPrim and AnevPrim SMCs.

Tab. S3 P-values characterizing the significance between two populations of the boxplot data showing the distribution of the measured traction forces (TF) (experimental data) for AoPrim and AnevPrim lineages for each gel stiffness. Significance between two populations was assessed using a Mann-Whitney test. Significance was stated there for $p<5\%$. P-values below this threshold are written in red.

Fig. S3 Histograms showing the distribution of the measured traction forces (TF) (experimental data) for AoPrim1 (a) and AnevPrim1 (b) lineages, and for each gel stiffness: 4, 8, 12, and 25 kPa (width of each bar: 35 nN). The green vertical bar represents the median.

Fig. S4 Histograms showing the distribution of the measured traction forces (TF) (experimental data) for AoPrim2 (a) and AnevPrim2 (b) lineages, and for each gel stiffness: 4, 8, 12, and 25 kPa (width of each bar: 35 nN). The green vertical bar represents the median.

Fig. S5 Histograms showing the distribution of the measured traction forces (TF) (experimental data) for AoPrim3 (a) and AnevPrim3 (b) lineages, and for each gel stiffness: 4, 8, 12, and 25 kPa (width of each bar: 35 nN). The green vertical bar represents the median.

Fig. S6 Boxplots showing the distribution of the measured traction forces (TF) (experimental data) for AoPrim1 (a), AoPrim2 (b), and AoPrim3 (c) lineages, and for each gel stiffness: 4, 8, 12, and 25 kPa. Significance between two populations was assessed using a Mann-Whitney test. Significance was stated for p<5%. Only p-values below the 5% threshold are reported in the figure. Median values are also reported for a clearer comparison.

Fig. S7 Boxplots showing the distribution of the measured traction forces (TF) (experimental data) for AnevPrim1 (a), AnevPrim2 (b), and AnevPrim3 (c) lineages, and for each gel stiffness: 4, 8, 12, and 25 kPa. Significance between two populations was assessed using a Mann-Whitney test. Significance was stated for p<5%. Only p-values below the 5% threshold are reported in the figure. Median values are reported for a clearer comparison.

APPENDIX A.

SMC ARCHITECTURE AND MORPHOLOGY

Fig. S1

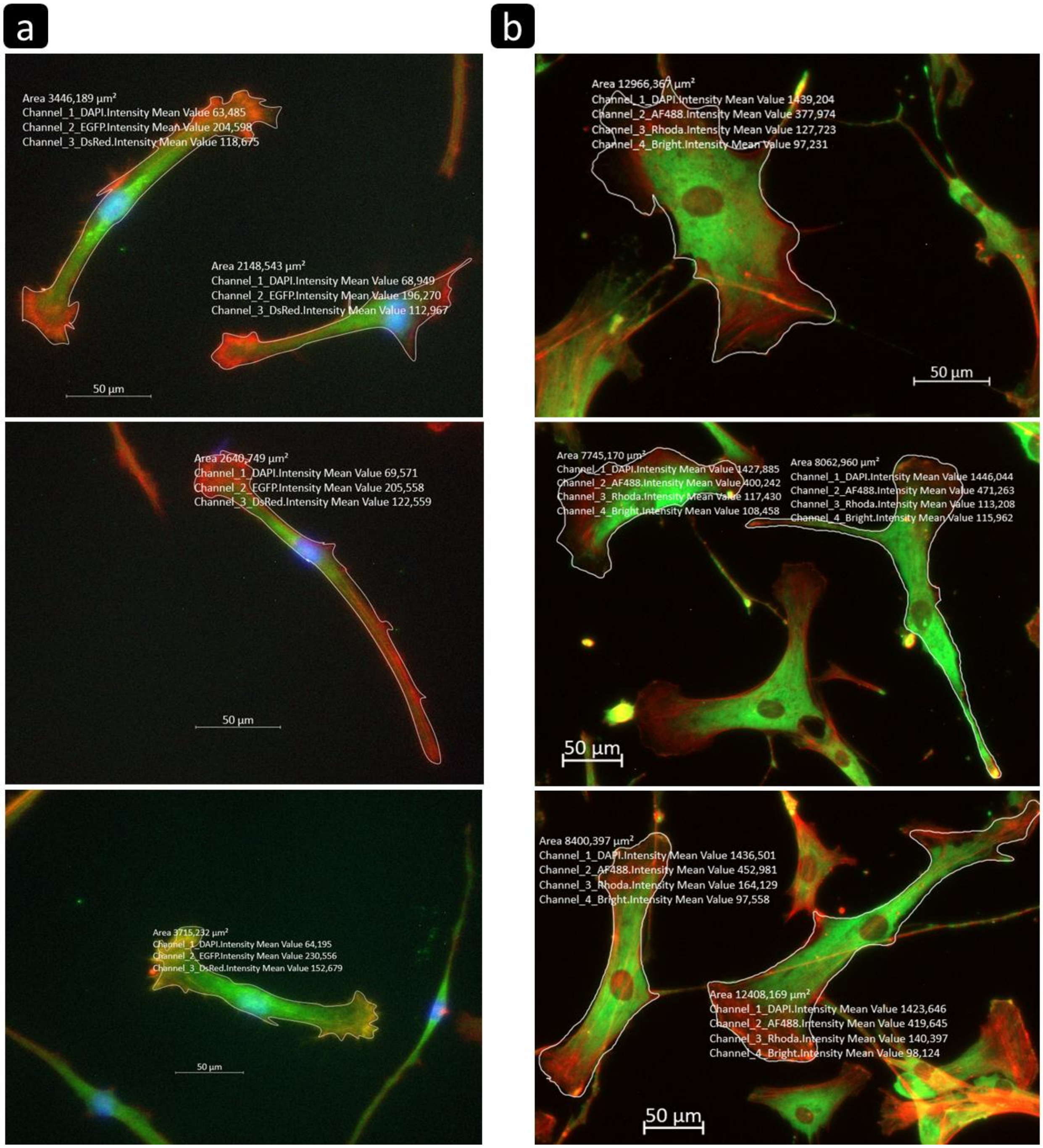
a
b
Area 3446,189 µm²
Channel_1_DAPI.Intensity Mean Value 63,485
Channel_2_EGFP.Intensity Mean Value 204,598
Channel_3_DsRed.Intensity Mean Value 118,675
Area 2148,543 µm²
Channel_1_DAPI.Intensity Mean Value 68,949
Channel_2_EGFP.Intensity Mean Value 196,270
Channel_3_DsRed.Intensity Mean Value 112,967
50 µm
Area 2640,749 µm²
Channel_1_DAPI.Intensity Mean Value 69,571
Channel_2_EGFP.Intensity Mean Value 205,558
Channel_3_DsRed.Intensity Mean Value 122,559
50 µm
Area 3715,232 µm²
Channel_1_DAPI.Intensity Mean Value 64,195
Channel_2_EGFP.Intensity Mean Value 230,556
Channel_3_DsRed.Intensity Mean Value 152,679
50 µm
Area 12966,367 µm²
Channel_1_DAPI.Intensity Mean Value 1439,204
Channel_2_AF488.Intensity Mean Value 377,974
Channel_3_Rhoda.Intensity Mean Value 127,723
Channel_4_Bright.Intensity Mean Value 97,231
50 µm
Area 7745,170 µm²
Channel_1_DAPI.Intensity Mean Value 1427,885
Channel_2_AF488.Intensity Mean Value 400,242
Channel_3_Rhoda.Intensity Mean Value 117,430
Channel_4_Bright.Intensity Mean Value 108,458
Area 8062,960 µm²
Channel_1_DAPI.Intensity Mean Value 1446,044
Channel_2_AF488.Intensity Mean Value 471,263
Channel_3_Rhoda.Intensity Mean Value 113,208
Channel_4_Bright.Intensity Mean Value 115,962
50 µm
Area 8400,397 µm²
Channel_1_DAPI.Intensity Mean Value 1436,501
Channel_2_AF488.Intensity Mean Value 452,981
Channel_3_Rhoda.Intensity Mean Value 164,129
Channel_4_Bright.Intensity Mean Value 97,558
Area 12408,169 µm²
Channel_1_DAPI.Intensity Mean Value 1423,646
Channel_2_AF488.Intensity Mean Value 419,645
Channel_3_Rhoda.Intensity Mean Value 140,397
Channel_4_Bright.Intensity Mean Value 98,124
50 µm

Fig. S2

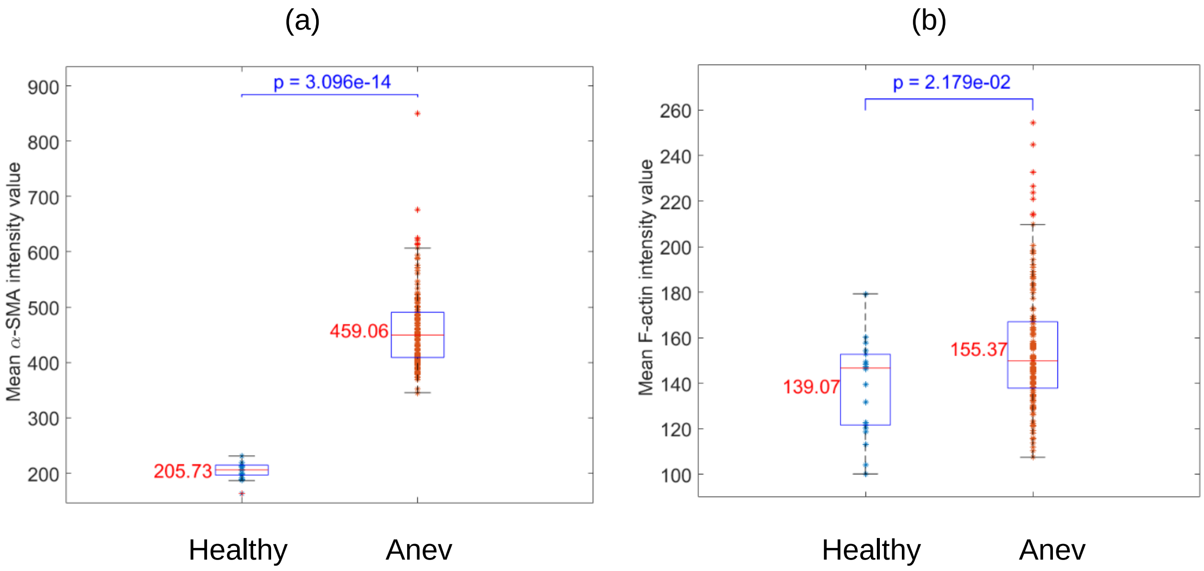
(a)
p = 3.096e-14
Mean α-SMA intensity value
900
800
700
600
500
400
300
200
459.06
205.73
Healthy
Anev
(b)
p = 2.179e-02
Mean F-actin intensity value
260
240
220
200
180
160
140
120
100
155.37
139.07
Healthy
Anev

APPENDIX B.

FULL TFM DATA

ON 24-WELL PLATES

Tab. S1

Stiffness	4 kPa	8 kPa	12 kPa	25 kPa	ΔF
**AoPrim**					
AoPrim 1	55.6 nN	78.0 nN	95.1 nN	100.4 nN	418.3 nN
AoPrim 2	54.9 nN	46.8 nN	25.6 nN	70.2 nN	262.8 nN
AoPrim 3	51.9 nN	74.7 nN	74.7 nN	80.0 nN	341.9 nN
**AnevPrim**					
AnevPrim 1	44.7 nN	49.8 nN	80.3 nN	103.1 nN	454.1 nN
AnevPrim 2	112.2 nN	126.7 nN	155.9 nN	120.6 nN	774.0 nN
AnevPrim 3	116.6 nN	215.1 nN	383.2 nN	208.5 nN	843.9 nN

Tab. S2

	4 kPa	8 kPa	12 kPa	25 kPa
**AoPrim**				
AoPrim1				
**F < 60 nN**	68.8 %	31.3 %	52.9 %	47.1 %
**F > 200 nN**	6.2 %	0 %	11.8 %	5.9 %
AoPrim2				
**F < 60 nN**	69.6 %	68.2 %	90.9 %	50.0 %
**F > 200 nN**	0 %	4.6 %	0 %	0 %
AoPrim3				
**F < 60 nN**	75.0 %	50.0 %	43.5 %	50.0 %
**F > 200 nN**	4.2 %	4.5 %	4.4 %	0 %
**AnevPrim**				
AnevPrim1				
**F < 60 nN**	81.5 %	75.0 %	53.7 %	42.9 %
**F > 200 nN**	7.4 %	5.0 %	7.3 %	14.3 %
AnevPrim2				
**F < 60 nN**	16.2 %	16.0 %	27.3 %	28.9 %
**F > 200 nN**	13.5 %	16.0 %	29.5 %	15.8 %
AnevPrim3				
**F < 60 nN**	27.1 %	8.9 %	1.8 %	4.4 %
**F > 200 nN**	20.8 %	42.2 %	75.9 %	40.0 %

Tab. S3

a

4kPa	Ao1	Ao2	Ao3	Anev1	Anev2	Anev3
**Ao1**	1	**0.79**	**0.77**	0.26	4.37E-04	5.30E-04
**Ao2**		1	**0.94**	0.17	3.14E-04	1.62E-04
**Ao3**			1	7.30E-02	5.57E-05	2.83E-05
**Anev1**				1	**2.24E-06**	**1.32E-07**
**Anev2**					1	**0.80**
**Anev3**						1

b

8kPa	Ao1	Ao2	Ao3	Anev1	Anev2	Anev3
**Ao1**	1	**1.25E-02**	**0.56**	1.23E-02	7.91E-03	1.21E-04
**Ao2**		1	**5.00E-02**	0.72	5.21E-07	3.64E-08
**Ao3**			1	1.82E-02	1.90E-03	4.27E-05
**Anev1**				1	**2.86E-08**	**1.38E-10**
**Anev2**					1	**3.24E-03**
**Anev3**						1

c

12kPa	Ao1	Ao2	Ao3	Anev1	Anev2	Anev3
**Ao1**	1	**8.74E-04**	**0.85**	0.68	2.48E-02	5.19E-07
**Ao2**		1	**2.93E-04**	3.04E-04	3.41E-09	1.46E-11
**Ao3**			1	0.83	1.24E-03	5.48E-10
**Anev1**				1	**5.39E-04**	**6.27E-13**
**Anev2**					1	**2.69E-08**
**Anev3**						1

d

25kPa	Ao1	Ao2	Ao3	Anev1	Anev2	Anev3
**Ao1**	1	**0.43**	**0.80**	0.89	0.15	2.36E-04
**Ao2**		1	**0.47**	0.55	4.12E-03	3.77E-07
**Ao3**			1	1	3.88E-02	1.05E-06
**Anev1**				1	**3.79E-02**	**3.18E-06**
**Anev2**					1	**3.97E-04**
**Anev3**						1

Fig. S3

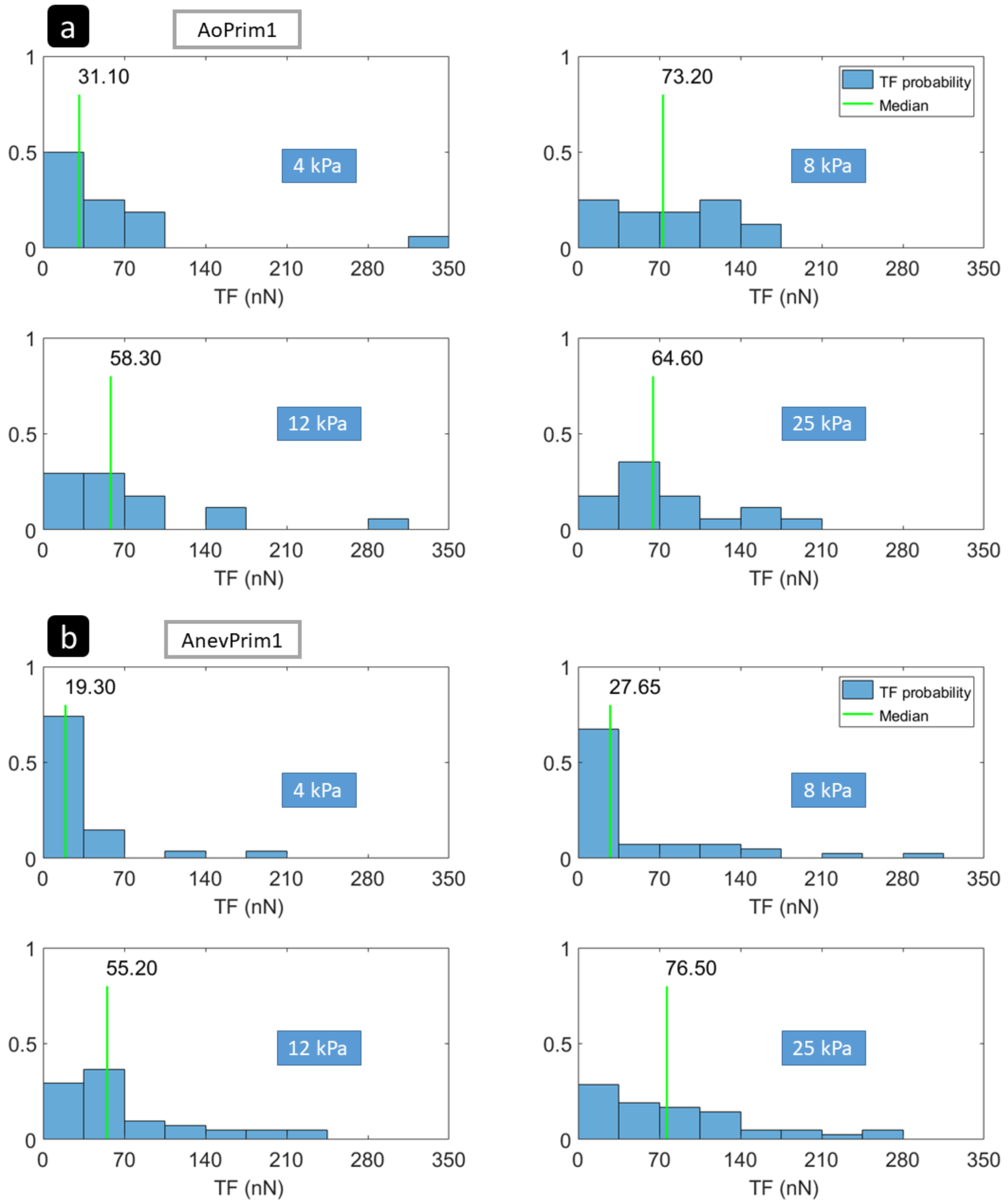
a
AoPrim1
31.10
4 kPa
73.20
TF probability
Median
8 kPa
58.30
12 kPa
64.60
25 kPa
b
AnevPrim1
19.30
4 kPa
27.65
TF probability
Median
8 kPa
55.20
12 kPa
76.50
25 kPa
0
0.5
1
0
70
140
210
280
350
TF (nN)

Fig. S4

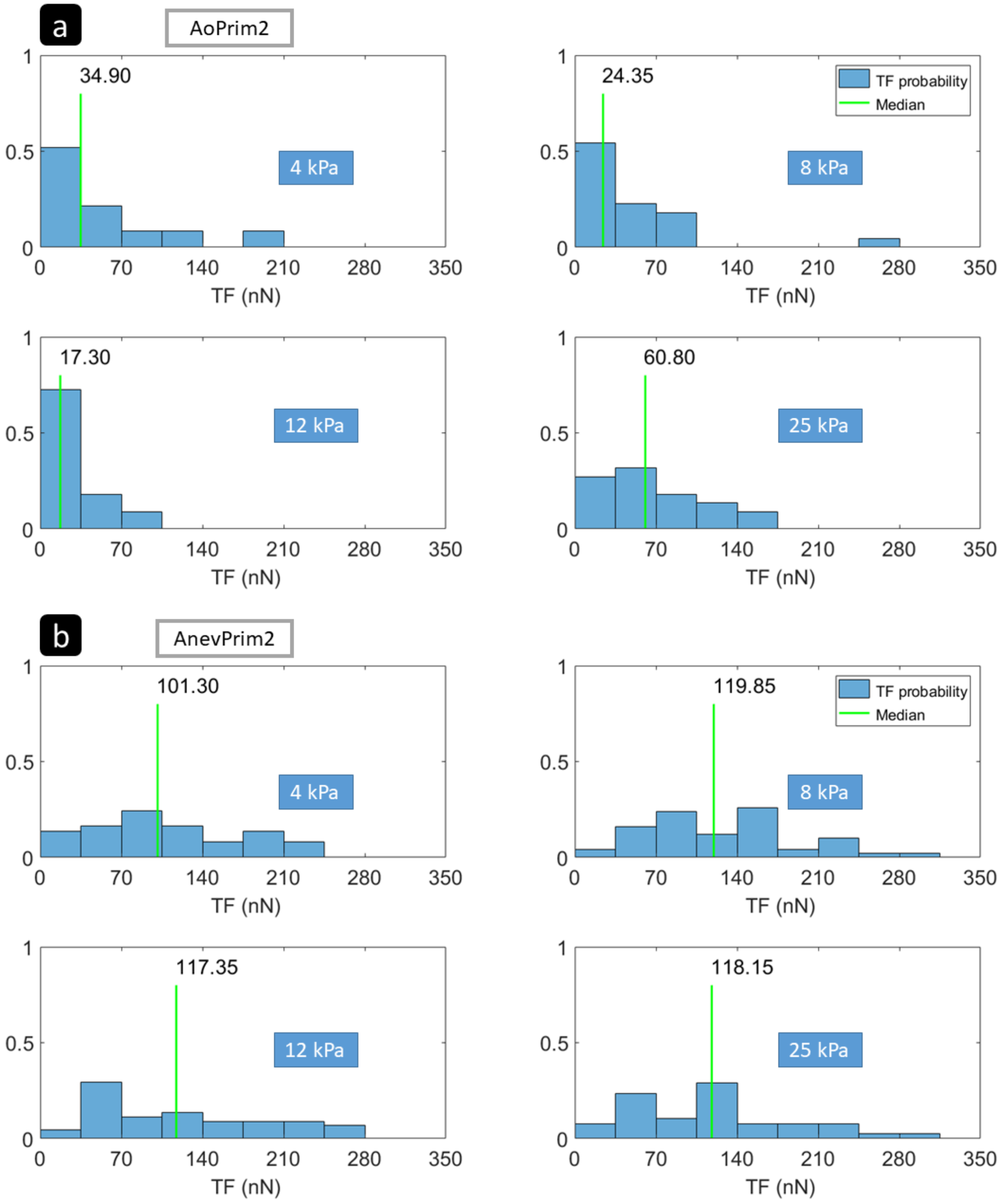
a
AoPrim2
34.90
4 kPa
24.35
8 kPa
TF probability
Median
17.30
12 kPa
60.80
25 kPa
b
AnevPrim2
101.30
4 kPa
119.85
8 kPa
TF probability
Median
117.35
12 kPa
118.15
25 kPa
TF (nN)
0
70
140
210
280
350
0
0.5
1

Fig. S5

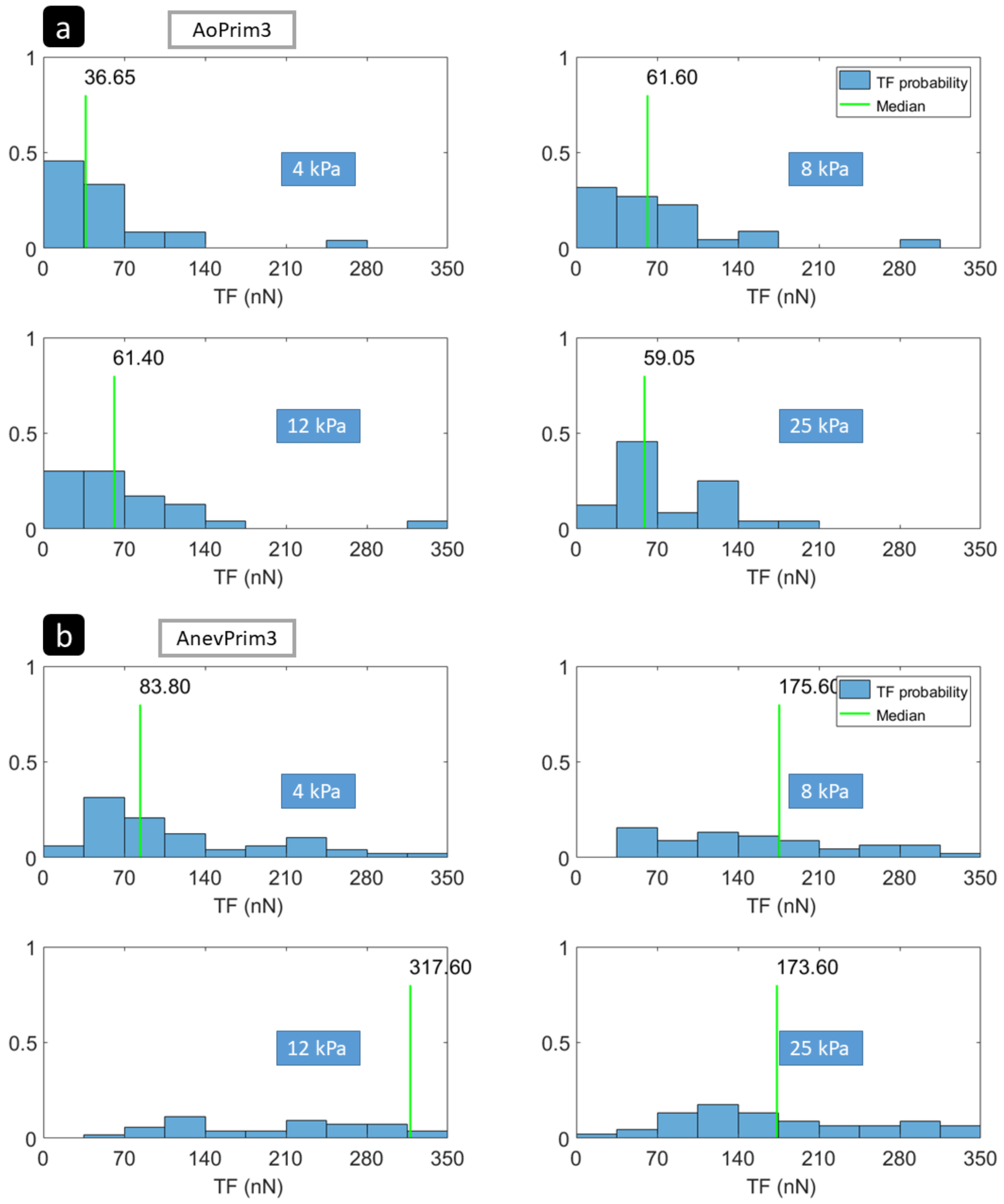

a
AoPrim3
36.65
4 kPa
61.60
TF probability
Median
8 kPa
61.40
12 kPa
59.05
25 kPa
b
AnevPrim3
83.80
4 kPa
175.60
8 kPa
317.60
12 kPa
173.60
25 kPa
TF (nN)
0
70
140
210
280
350
0
0.5
1

Fig. S6

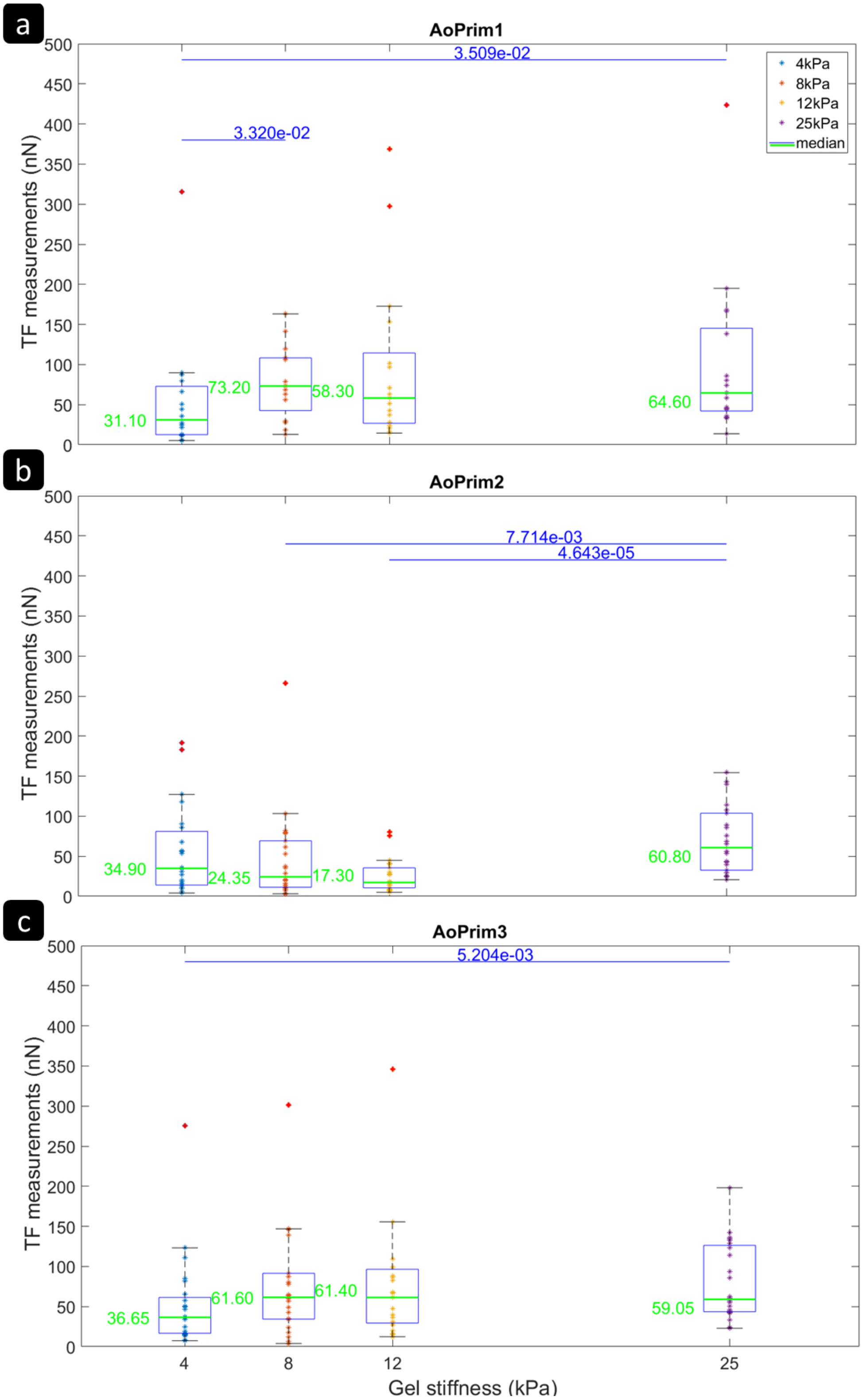
a
AoPrim1
3.509e-02
3.320e-02
4kPa
8kPa
12kPa
25kPa
median
31.10
73.20
58.30
64.60
TF measurements (nN)
b
AoPrim2
7.714e-03
4.643e-05
34.90
24.35
17.30
60.80
TF measurements (nN)
c
AoPrim3
5.204e-03
36.65
61.60
61.40
59.05
TF measurements (nN)
Gel stiffness (kPa)

Fig. S7

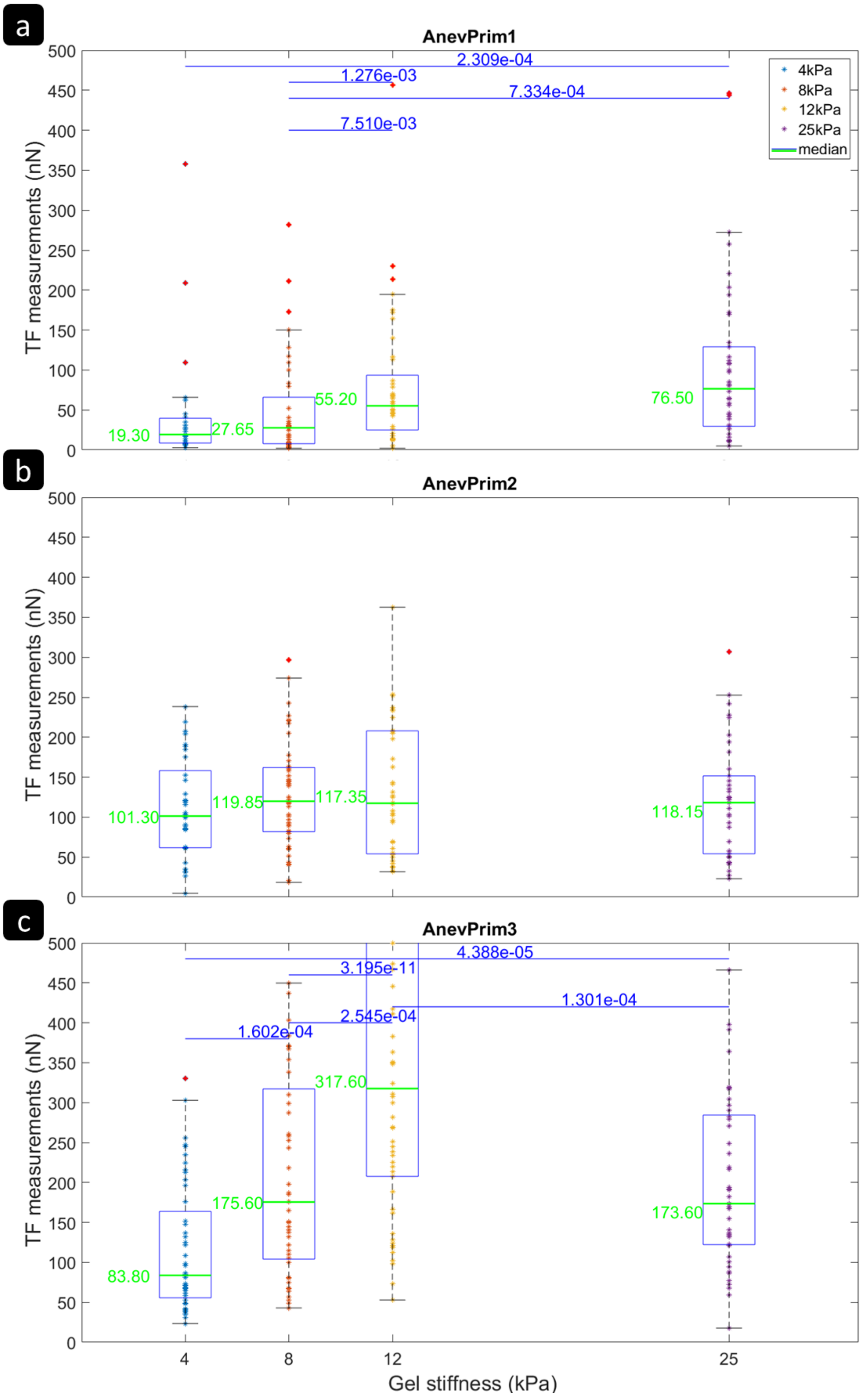
a
AnevPrim1
2.309e-04
1.276e-03
7.334e-04
7.510e-03
4kPa
8kPa
12kPa
25kPa
median
19.30
27.65
55.20
76.50
b
AnevPrim2
101.30
119.85
117.35
118.15
c
AnevPrim3
4.388e-05
3.195e-11
1.301e-04
2.545e-04
1.602e-04
317.60
175.60
173.60
83.80
TF measurements (nN)
Gel stiffness (kPa)
4
8
12
25
